# Amentoflavone Induces Autophagy and Modulates p53

**DOI:** 10.22074/cellj.2019.5717

**Published:** 2018-11-18

**Authors:** Hye-Jung Park, Moon-Moo Kim

**Affiliations:** 1 Department of Chemistry and Biology, Dong-Eui University, Busan, Republic of Korea; 2 Department of Applied Chemistry, Dong-Eui University, Busan, Republic of Korea

**Keywords:** Aging, Amentoflavone, Autophagy, p53, SIRT1

## Abstract

**Objective:**

Amentoflavone is the main component of Selaginella tamariscina widely known as an oriental traditional medicinal stuff that has been known to have a variety of medicinal effects such as the induction of apoptosis, anti- metastasis, and anti-inflammation. However, the effect of amentoflavone on autophagy has not been reported until now. The aim of this study was to investigate whether amentoflavone has a positive effect on the induction of autophagy related to cell aging.

**Materials and Methods:**

In this experimental study, the aging of young cells was induced by the treatment with insulin- like growth factor-1 (IGF-1) at 50 ng/mL three times every two days. The effect of amentoflavone on the cell viability was evaluated in A549 and WI-38 cells using 3-(4,5-dimethyl-2-yl)-2,5- diphenyl tetrazolium bromide (MTT) assay. The induction of autophagy was detected using autophagy detection kit. The expression of proteins related to autophagy and IGF-1 signaling pathway was examined by western blot analysis and immunofluorescence assay.

**Results:**

First of all, it was found that amentoflavone induces the formation of autophagosome. In addition, it enhanced the expression level of Atg7 and increased the expression levels of Beclin1, Atg3, and LC3 associated with the induction of autophagy in immunofluorescence staining and western blot analyses. Moreover, amentoflavone inhibited the cell aging induced by IGF-1 and hydrogen peroxide. In particular, the levels of p53 and p-p21 proteins were increased in the presence of amentoflavone. Furthermore, amentoflavone increased the level of SIRT1 deacetylating p53.

**Conclusion:**

Our results suggest that amentoflavone could play a positive role in the inhibition of various diseases associated with autophagy and the modulation of p53.

## Introduction

Amentoflavone used in the study is mainly contained in *Selaginella tamariscina* to have a hemostatic effect, anti-inflammatory and anti-cancer effect (1). *Selaginella tamariscina* has been used as an anti-cancer agent and contains many different compounds such as biflavonoids (2) which are widely present in vascular plants and have a variety of physiological activities (3, 4). Amentoflavone is a dimer composed of apigenin that has the capability to promote the cell cycle arrest and induction of apoptosis through the p53-related pathway as well as the induction of autophagy in several human cancer cell lines (5). However, the role of amentoflavone in the mechanism of the induction of autophagy remains unclear. 

Anti-aging studies have focused on manipulation of genes involved in histone acetylation, Insulin-like growth factor-1 (IGF-1) pathway, and p53 system to suppress the senescence as a mean to extend the lifespan of the mammalian model (6, 7). However, the efforts increasing longevity in complex animal models do not have a sufficient understanding of the life mechanism. On the other hand, p53, a tumor suppressor protein, is closely related to aging as well as the induction of autophagy and apoptosis. In particular, the activated IGF-1 signaling is involved in the senescence and cell growth via p53 protein dependently. The short-term of IGF-1 treatment promotes the cell growth by up-regulating PI3K/AKT pathway through IGF1R against p53 protein. In contrast, the long-term IGF-1 treatment induces the senescence and is also very closely related to the development of cancer, depending on the concentration of the p53 protein that is as a substrate for SIRT1, a histone deacetylase, resulting in the inhibition of cell aging caused by long-term IGF-1 treatment (8, 9). 

In recent years, resveratrol or spermidine, calorie restriction, and rapamycin have been reported to induce autophagy associated with longevity (10). Therefore, it is important to study the relationship and the mechanism of senescence related to IGF-1, p53, and HAT/SIRT1 pathway associated with the induction of autophagy to remove the cellular wastes such as organelles and macromolecules damaged by internal and external stimuli. In addition, the previous study reported that there is substantial evidence supporting the roles of autophagy in megakaryopoiesis. The engagement of transcription factors, cytokines, and extracellular stress synergically promotes the maturation of megakaryocytes (11). Transcription factors, such as SCL, GATA1, GATA2, and NF-E2 allow the development of megakaryocyte/erythroid progenitor cells (12). The abrogation of autophagy from stem cell stage by hematopoietic knockout of ATG7 leads to impaired megakaryopoiesis, the loss of autophagy caused mitochondrial and cell cycle dysfunction, impeding megakaryopoiesis, and megakaryocyte differentiation (13). 

While the active autophagy process prolongs the cell survival and lifespan, the over-activated autophagy leads to autophagic cell death (14). In the autophagic process, Beclin1 (Atg6) in the initial formation of autophagosome acts as a partner of Bcl-2 as an antiapoptosis factor that exerts an anti-autophagic effect as well as anti-apoptosis (15). Recently, some studies in breast cancer cell line confirmed that the expression level of Beclin1 is remarkably low and induces the tumor activity of cells (16). 

Accordingly, in this study, we investigated whether amentoflavone could modulate autophagy related to cell aging through the modulation of p53 and SIRT1. 

## Materials and Methods

This experimental study was conducted at the Department of Applied Chemistry at Dong-Eui University (Republic of Korea). In this study, Amentoflavone was obtained from Sigma-Aldrich (St. Louis, MO, USA). Dulbecco’s modified Eagle’s medium (DMEM), trypsin-EDTA, penicillin/ streptomycin/ amphotericin (10000 U/ml, 10000 g/ ml, and 2500 g/ml, respectively), and fetal bovine serum (FBS) were obtained from Gibco BRL, Life Technologies (NY, USA). A549 (ATCC # CRL-6323) and WI38 (ATCC # CRL-75) cells were purchased from ATCC. 3-(4,5-dimethyl-2-yl)-2,5- diphenyl tetrazolium bromide (MTT) reagent, agarose, and other materials were purchased from Sigma Chemical Co. (St. Louis, MO, USA). 

### Cell line and culture 

This project was approved by the Ethics Committee of Dong-Eui University of Applied Chemistry, Busan, Republic of Korea. Cell lines were separately grown as monolayers at 5% CO_2_ at 37°C in the humidified atmosphere using appropriate media supplemented with 5% FBS, 2 mM glutamine, and 100 g/ml penicillin- streptomycin. DMEM was used as the culture medium for A549 cells. Cells were passaged 3 times a week by treating with trypsin-EDTA.

### MTT assay

Cytotoxic levels of amentoflavone were measured using MTT method as described previously by Hansen et al. (17). The viability of cells was quantified as a percentage compared to the control (optical density of treated cells/optical density of blank×100) and dose-response curves were developed. The data were expressed as the mean from at least three independent experiments and P<0.05 was considered significant.

### Autophagosome detection assay 

Autophagy activity was detected using a commercially available autophagy/cytotoxicity dual staining kit from Cayman Chemical Company (Item No. 600140). Autophagy assay was performed according to the manufacturer’s protocol. In brief, A549 cells were seeded in a 96-well plate at a density of 5×10^4^ cells/well in DMEM culture medium and incubated overnight at 37°C. Then, cells were treated with different concentrations of amentoflavone and tamoxifen as a positive control and incubated overnight. On the third day, cells were stained with propidium iodide (PI) and monodansylcadaverine (MDC) according to the manufacturer’s protocol. Autophagic vacuole staining intensity can be detected using an excitation wavelength of 335 nm and an emission wavelength of 512 nm using microplate reader (Tecan Austria GmbH, Austria). The cells were also analyzed by fluorescent microscopy according to the manufacturer’s protocol. Dead cells are stained by propidium iodide and can be detected with a rhodamine filter (excitation/emission=540/570 nm).

### Analyses of proteins expression using western blot

Western blotting was performed according to the standard procedures. Cells treated with different concentrations of amentoflavone were lysed with RIPA lysis buffer (Sigma Chemical Co., St. Louis, MO, USA). The cell lysates were resolved on a 4-20% Novex®gradient gel (Invitrogen, USA), electrotransferred onto a nitrocellulose membrane and blocked with 10% skim milk. The primary antibodies (1:1,000) including p-p21(sc-12902, Santa Cruz Biotechnology., CA, USA), p53(sc-126X), p-p53(9286S, Sell Signaling Technology, MA, USA), ac-p53(06-758, Upstate Biotechnology Inc., NY 12946 USA), Atg3 (sc-393623), Atg7 (2631S, Sell Signaling), Beclin1 (3738, Sell Signaling), LC-3 (sc-292354), Bcl2 (sc-492-G), ß-actin (sc-1616), and their secondary antibodies (1:5,000) (sc-1616, sc-2354, sc-2005, Santa Cruz Biotechnology, CA, USA) were used to detect the respective proteins using a chemiluminescent ECL assay kit (Amersham Pharmacia Biosciences, NJ, USA) according to the manufacturer’s instructions. Protein bands were visualized using AlphaEase®gel image analysis software (Alpha Innotech, CA, USA) and protein expression was quantified by Multi Gauge V3.0 software (Fujifilm Life Science, Japan). 

### Analysis of protein expression using immunofluorescence staining 

Cells were seeded onto a slide chamber and were incubated overnight at 37°C. Then, the cells were treated with different concentrations of amentoflavone. After 24 hours of incubation, cells were fixed with 10% formalin for 15 minutes at room temperature followed by the permeabilization with phosphate buffer solution (PBS) containing 0.5% tween 20 (0.5% PBS T-20) and washed three times by 0.1% PBS T-20. The cells had preconditioning process with 5% Donkey normal serum and immunofluorescence staining with primary antibodies (anti-Atg7, anti-p53, anti-p-p21, anti-pmTOR) (1:500) for 24 hours at room temperature. After, the cells were then washed with 0.1% PBS T-20 three times for 5 minutes, respectively and treated with the secondary antibodies (donkey anti-rabbit conjugated CY3, donkey anti-goat conjugated CY3, donkey anti-goat conjugated FITC, donkey anti-rabbit conjugated CY3, donkey anti-goat conjugated FITC, donkey anti-mouse conjugated FITC, donkey anti-rabbit conjugated CY3) (CY3 1:400, FITC 1:200) at room temperature for 1 hour. The cells were then washed with 0.1% PBS T-20 three times and PB once for 5 minutes, respectively. Finally, the slide was spread using DAPI solution and examined using iRiS™ Digital Cell Imaging System (Logos Biosystems, Annandale, US). 

### SA-ß-galactosidase staining 

According to the method of Tran (8), WI38 and A549 cells were incubated in a 24-well plate format under a serum starvation state for 4 days, then exposed to 5 ng/mL of IGF-1 treatment for 6 days as a long-term treatment or 10 µM H_2_O_2_ treatment for 24 hours. After the treatment for 24 hours with a proper concentration of amentoflavone, the cell culture medium was aspirated and the cells were twice washed with PBS. After the last rinse, PBS was replaced with 250 µl of 4% paraformaldehyde (PFA) for the fixation. The cells were incubated for 5 minutes at room temperature. The 4% PFA was aspirated and the cells were washed two times for 5 minutes each at room temperature with gentle shaking with 500 µl of PBS. Each well was exposed to 250 µl of SA-ß-gal staining solution. The cells were incubated in the dark in a 37°C incubator. The reaction was terminated when the cells were stained as blue-green. To terminate the reaction, the staining solution was aspirated and replaced with distilled water. The cells were washed for the second time with distilled water. After the last wash, 500 µl of distilled water was added to each well and the plate was observed under the microscope.

### Statistical analysis 

Data were analyzed using Student’s t test for paired data (comparison with the control group) and MEGFL. Data are represented as the mean of values ± S.D and obtained from three independent experiments (*P<0.05, **P<0.01, ***P<0.001).

## Results

### Effect of amentoflavone on the cytotoxicity 

**Fig.1:**
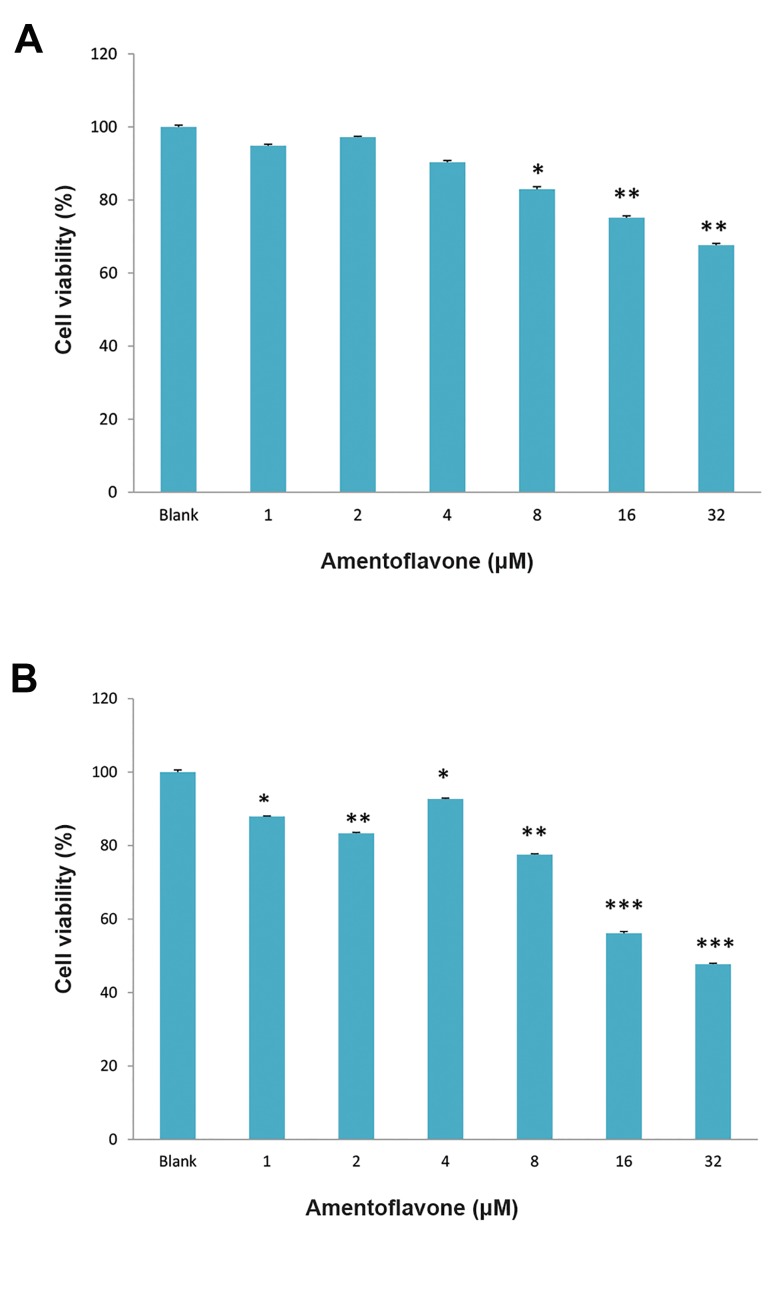
Effect of amentoflavone on cell viability. A. Amentoflavone was treated to A549 cells and B. Amentoflavone was treated to WI38 cells. The cells were treated with amentoflavone at the indicated concentration and the cell viability was determined by MTT assay after 48 hours. Data are presented as the mean of values ± SD obtained from three independent experiments. The level of significance was identified statistically (*; P<0.05, **; P<0.01, ***; P<0.001) using Student’s t test.

The cytotoxic effect of amentoflavone was investigated using MTT assay. Amentoflavone above 8 µM exhibited Park and Kim the cytotoxicity in cancerous human lung fibroblasts (A549 cells) as shown in Figure 1A. In addition, amentoflavone above 1 µM showed the cytotoxicity in normal human lung fibroblasts (WI38 cells) as shown in Figure 1B.

### Effect of amentoflavone on the formation of autophagosome

**Fig.2:**
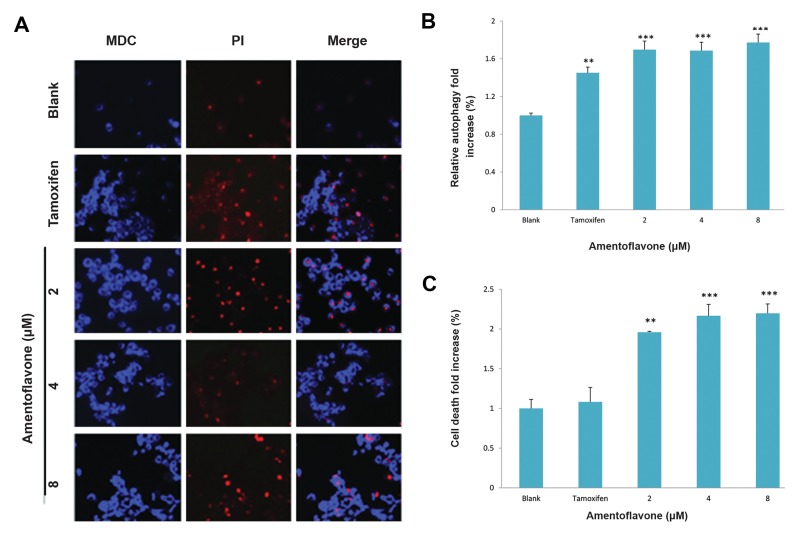
Effect of amentoflavone on formation of autophagosome in A549 cells. The cells (1×10^5^ cells) were treated with amentoflavone at the indicated concentration. The level of autophagosome formation was evaluated in the presence of amentoflavone or tamoxifen. A. The autophagosome was stained by MDC and the damaged cells or dying cells were stained by PI, B. The effect of amentoflavone on autophagy was analyzed by the fluorescence measurement of autophagic vacuole. The cells showing autophagic vacuoles were quantified by fold increase in green detection reagent signal, and C. The effect of amentoflavone on the cell viability was analyzed by the fluorescence measurement of dead cells stained by propidium iodide. Data are shown as the mean of values ± SD obtained from three independent experiments. The level of significance was identified statistically (**; P<0.01, ***; P<0.001) using Student’s t test. MDC; Monodansylcadaverine and PI; propidium iodide.

The effect of amentoflavone on the formation of autophagosome was investigated by the degree of MDC absorbed into autophagosome by the action of autophagy. Amentoflavone above 2 µM displayed a remarkable fluorescence image in A549 cells as shown in Figure 2A. It was observed that it induced autophagy by 1.7-fold increase compared with tamoxifen treatment group used as a positive control in Figure 2B. In order to examine its cytotoxicity at the same time, the cells were stained with PI. Amentoflavone above 2 µM showed PI staining that means the cell death as shown in Figure 2C.

### Effect of amentoflavone on the expression of Atg7 and autophagy-related proteins

**Fig.3:**
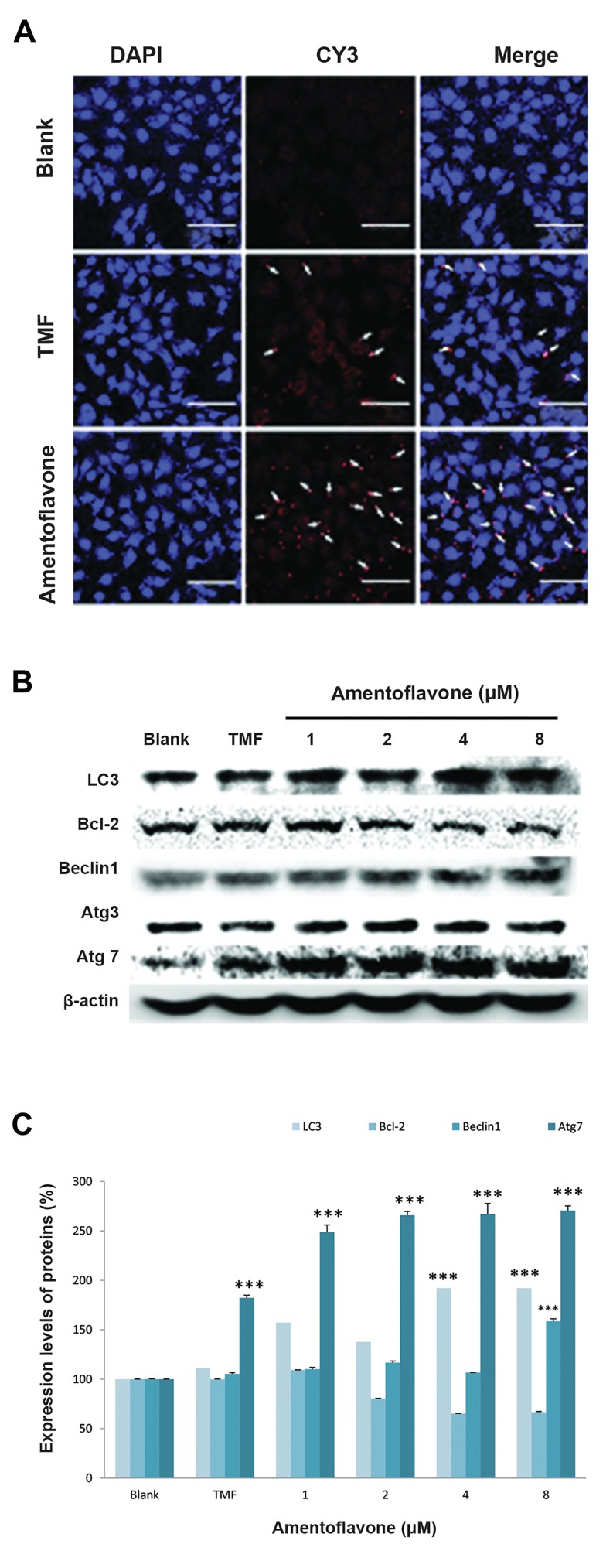
Effect of amentoflavone on the expression of Atg7 and autophagy- related proteins. A. The images of Atg7 immunofluorescence-stainedA549 cells were shown by the red color. The arrows show Atg7 hasbeen localized to the cell cytosol (scale bar: 100 µm), B. The effect of amentoflavone on protein expressions of LC3, Bcl-2, Beclin1, Atg3, Atg7, and ß-actin was analyzed by western blot, and C. The level of proteins expression was quantified by Multi Gauge V3.0 software. Dataare presented as the mean of values ± SD from three independentexperiments. The level of significance was identified statistically (***; P<0.001) using Student’s t test.

In order to confirm the effect of amentoflavone on the induction of autophagy, the expression of Atg7, an autophagic marker, was examined in A549 cells using immunofluorescence analysis. Cell nuclei were labeled with blue DAPI fluorescence dye and the target protein Atg7 was stained with a red CY3 fluorescence dye as shown in Figure 3A. It was confirmed that amentoflavone at 4 µM exhibited a higher red image than tamoxifen at 20 µM used as a positive control, indicating that amentoflavone could induce a higher expression of Atg7 protein related to induction of autophagy. Western blotting was also carried out to examine the level of autophagy-related proteins by amentoflavone. Amentoflavone at 8 µM remarkably increased the levels of LC3, Becline1, and Atg7 involved in the formation of autophagosome in A549 cells as shown in Figure 3B and 3C. Moreover, it was found that it can increase the levels of above-mentioned proteins at a higher level than the tamoxifen group. However, amentoflavone decreased the level of Bcl-2 protein, an anti-autophagy marker, and did not affect the level of Atg3. 

### Effect of amentoflavone on the expression of proteins related to p53 signaling pathway

**Fig.4:**
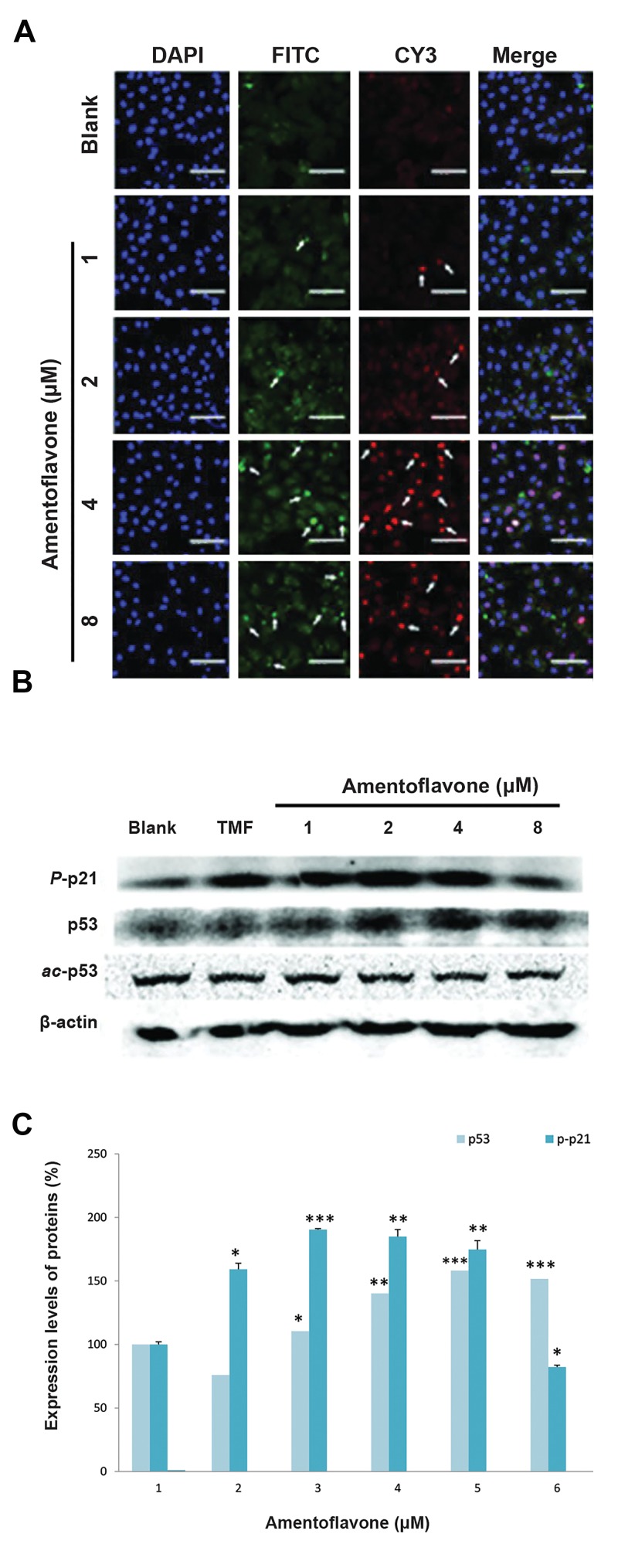
Effect of amentoflavone on the expression of proteins related to p53 signaling pathway. A. The images of p53 (FITC) and p-p21 (CY3) immunofluorescence-stained A549 cells were shown by the green and red color, respectively. Arrows show p53 and p-p21 have been localized to the cell cytosol or nuclear (scale bar: 100 µm), B. The effect of amentoflavone on protein expressions of p-p21, p53, ac-p53, and p-p53 was analyzed by western blot, and C. The level of protein expressions was quantified by Multi Gauge V3.0 software.

In order to investigate the effect of amentoflavone on p53 signaling pathway involved in the senescence mechanism, immunofluorescence for p53 and p21 proteins were performed in this study. The nucleus of A549 cells was stained with DAPI, and the p-p21 and p53 proteins were labeled with green FITC and red CY3 fluorescence dyes, respectively. Amentoflavone treatment above 2 µM or 4 µM remarkably increased the levels of p53 protein and p-p21 proteins, respectively, as shown in Figure 4A. The effect on the level of proteins related to p53 signaling pathway was examined using western blot analysis. Amentoflavone above 2 µM increased the level of p53 as shown in Figure 4B and C. Similarly, the level of p-p21 protein was enhanced in the presence of amentoflavone above 2 µM. However, amentoflavone above 2 µM decreased the level of acetyl-p53. 

### Immunofluorescence analysis for the effect of amentoflavone on expression of SIRT1

**Fig.5:**
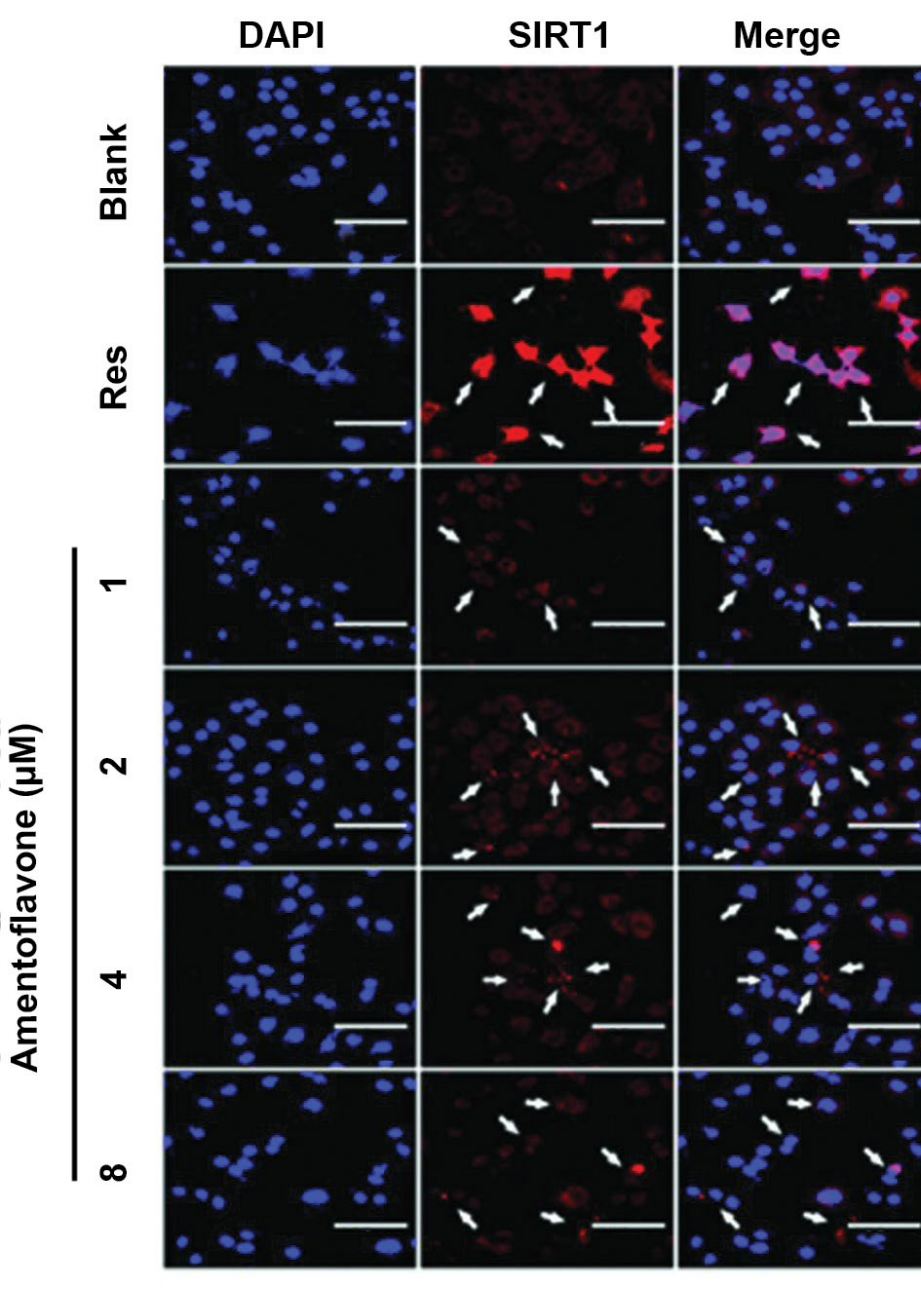
Immunofluorescence analysis of the effect of amentoflavone on expression of SIRT1. The images of SIRT1 (CY3) immunofluorescencestained A549 cells were shown by the red color. The cells were cultured in the presence of amentoflavone and detected with a rabbit polyclonal antibody against SIRT1. Arrows show SIRT1 has been localized to the cell cytosol. Res stands for resveratrol used as a positive control in this study (scale bar: 100 µm).

The anti-aging effect of amentoflavone was investigated by the analysis of SIRT1 protein expression involved in the senescence mechanism using immunofluorescence staining. The nucleus of the cell was stained with DAPI, and the SIRT1 protein was labeled with a red CY3 fluorescence dye, respectively. Resveratrol treatment used as a positive control showed the highest level of SIRT1 protein in the treatment groups as shown in Figure 5. Although the effect of amentoflavone on the level of SIRT1 was lower than that of the resveratrol treatment group, it remarkably increased the level of SIRT1 compared with the blank group. 

### Effect of amentoflavone on cell aging in A549 cells and WI38 cells 

**Fig.6:**
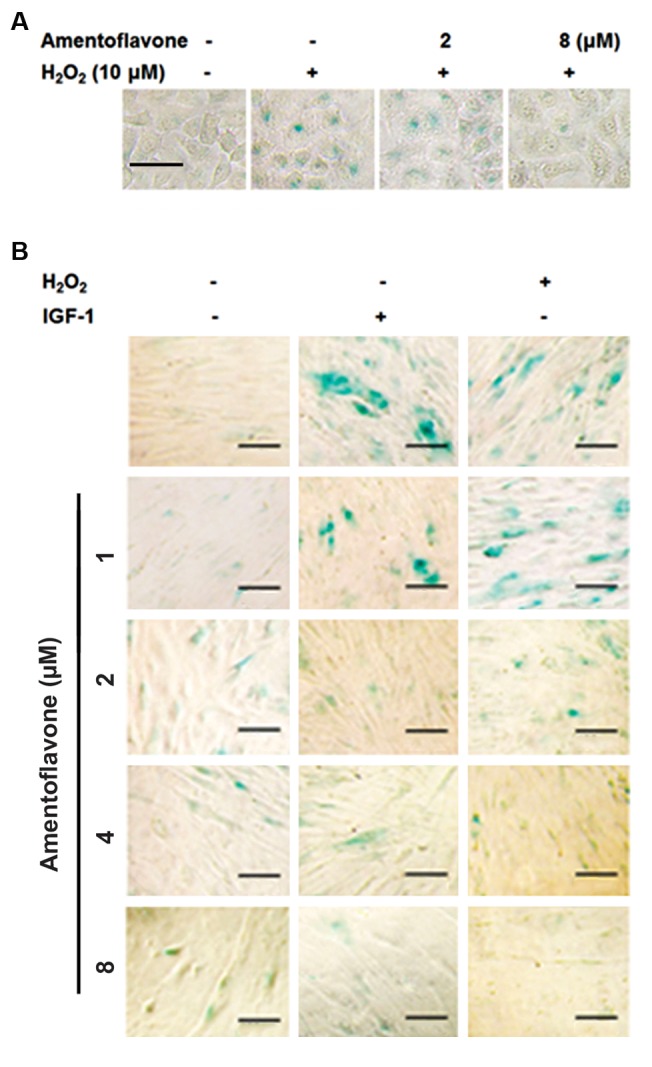
Effect of amentoflavone on senescence-associated (SA)-ß-galactosidase staining in A549 cells and in human lung fibroblast cells (WI38). A. After the cells were treated with amentoflavone and H_2_O_2_ (10 µM) for 24hours, SA-ß-gal staining was carried out (scale bar: 100 µm). The senescentcells were stained by the blue color and B. After the cells were treated with amentoflavone and IGF-1 (5 ng/mL) for 6 days or H_2_O_2_ (10 µM) for 24 hours, SA-ß-gal staining assay was carried out (scale bar: 100 µm).

The effect of amentoflavone on aging of A549 cells was investigated using SA-ß-galactosidase staining assay as a senescence marker. In this study, the aging of cells was induced by the short-term of treatment with H_2_O_2_ at 10 µM. H_2_O_2_ treatment showed a higher blue staining image than the blank group, indicating that H_2_O_2_ can induce the cell aging as shown in Figure 6A. However, amentoflavone treatment remarkably decreased the blue staining image induced by H_2_O_2_, indicating that the cell aging induced by H_2_O_2_ is inhibited by amentoflavone. The effect of amentoflavone on senescence was also examined using SA-ß-galactosidase staining assay normal lung fibroblasts (WI38 cells). The senescence of WI38 cells was induced by the long-term treatment of 5 ng/mL of IGF-1 or the treatment with H_2_O_2_ at 10 µM. The IGF-1 treated group exhibited a good phenotype of the cellular senescence showing a strong blue staining with a wide flattened shape, a typical shape of the aged cell as shown in Figure 6B. H_2_O_2_treated group showed the phenotype of the cellular senescence but the shape of the aged cell was slightly less than the IGF-1 treated group. It was observed that the amentoflavone treatment group above 2 µM reduced the degree of blue staining and increased the cell size into the wide flattened shape, indicating that amentoflavone reduces the senescence. 

## Discussion

As various studies on longevity have progressed, a variety of pathways associated with aging have been found and some gene manipulations succeed the life extension of the simple model such as the nematode (18) .In recent years, autophagy is closely related to the aging. Fasting related to IGF-1 pathway and rapamycin associated with mTOR mechanism necessarily require autophagy process for the life extension (19). Therefore, this study focused on the investigation whether the inductive effect of amentoflavone, a biflavonoid compound contained in Selaginella tamariscina, on autophagy could modulate the senescence induced by the long-term of IGF-1 treatment via p53 and SIRT1 signaling pathway. 

In the first place, the effect of amentoflavone on the induction of autophagy was determined by the formation of autophagosome that is the first step in autophagy process. Beclin1 (Atg6) and class . phosphatidylinositol 3-kinase (PI3 kinase) form a complex with Vps34, creating inactive form of the autophagosome (20). Amentoflavone increased the formation of such autophagosome and, remarkably, also enhanced the level of Beclin1 in A549 cells. Amentoflavone exhibited a higher effect than tamoxifen used as a positive control in forming autophagosome. Amentoflavone is believed to promote the formation of the initial autophagosome by increasing not only the extension of the phagophore and the expression of LC3 (Atg8) protein promoting the formation of autophagosome but also the expression of Atg7 activating its ubiquitination. Ubiquitin-like protein of Atg8 exists in a complex form (Atg8-PE) with phosphatidylethanolamine (PE) in autophagic membranes (or phagophore) and mediates the fusion portion of liposomes containing Atg8PE and tethering in *in vitro* system (21). Therefore, the positive effect of amentoflavone on the expression of LC3 and Atg7 proteins could affect even after the formation autophagosome, and the effect of amentoflavone on a later stage of autophagy should be further studied. 

These findings confirm that amentoflavone strongly promotes the autophagy process in the early stage, in particular by increasing the formation of autophagosome than tamoxifen by increasing the level of the Beclin1, Atg7, and LC3 proteins. Moreover, a previous study reported that the crosstalk between autophagy and apoptosis can be modulated by the interaction between Bcl-2 family proteins and Beclin1, a Bcl-2 interacting protein that promotes autophagy (22).

On the other hand, it was previously reported that apigenin. The monomer of amentoflavone, inhibits mTOR, an autophagy repressor, and its downstream target p70S6K, but does not alter the level of Beclin1, (23). In addition, it was found that the induction of autophagy by apigenin-mediated AMPK activation is accompanied by the inhibition of the mTOR signaling pathway as a potent chemopreventive agent (24).

In this study, amentoflavone inhibited the protein expression of Bcl-2, which is consistent with the previous report that Bcl-2 not only acts as an anti-apoptosis factor but also functions as an anti-autophagy factor (25), indicating that amentoflavone could induce apoptosis. Amentoflavone decreased the level of Bcl-2 protein which inhibits the formation of Bcl-2-Beclin1, complex and promotes the dissociation of Beclin1, leading to the induction of autophagy as well as apoptosis which is promoted by the inhibition of Bcl-2 protein (26). At this point, the action mechanism of amentoflavone on the induction of autophagy is distinct from that of apigenin.

The previous studies have suggested that amentoflavone has a great development potential as an anti-cancer drug on apoptosis in this inductive effect (25, 27). Our findings also suggest that amentoflavone could be developed as a potential anti-cancer drug. Although p53 induces apoptosis, it is a tumor suppressor protein which is closely involved in the development of cancer (28). Moreover, p53 protein is closely related to the aging mechanism (29). SIRT1 and p53 proteins have been reported to play a key role in the senescence induced by the insulin-like growth factor-1 (8). The activity of SIRT1, suppressed by the treatment of IGF-1 in the long-term, reduces the deacetylation of p53, resulting in the induction of senescence. In another report, SIRT1 suppresses the senescence in normal cells such as HDF, but it induces the senescence in some cancer cells such as MCF-7 and H1299 by inhibiting their growth and proliferation (30, 31). Thus, SIRT1 is an important factor in determining the activity of p53 (8). The activation of p53 protein is made by the SIRT1, as a histone deacetylase, that directly deacetylates p53 protein (32). In this study, amentoflavone increased the level of p53 protein, matching the increase of p-p21 expression activated by the p53 transcription factor. However, the level of acetyl-p53 protein was decreased by amentoflavone.

Although the expression of p53 is increased by amentoflavone, the activation of the p53 protein by the increased SIRT1 is believed to be offset. Therefore, this result is explained to be caused by the increased expression of SIRT1. The previous studies on the interaction of SIRT1 with p53 protein support that amentoflavone increases the level of p53 protein, but rather its increase in SIRT1 level explains very well our result that it could induce autophagy higher than apoptosis (8, 33). Moreover, most of aging studies have reported that the accumulation of p53 protein causes the aging of cells, but the explanation on the aging of an organism is not sufficient yet by the accumulation of p53 protein alone. In fact, p53 protein inhibits the aging of cells, and the inhibitory effect of the cell aging by p53 protein disappears by nutrin3a, a p53 inhibitor (34). Finally, the study on the premature aging of WI38 cells induced by H_2_O_2_ and the long-term of IGF1 confirmed that amentoflavone could inhibit the aging of these cells. In this study, we investigated to observe the effect of amentoflavone on the induction of autophagy at the protein level, and how the induction of autophagy affects the aging of the cell. 

## Conclusion

Amentoflavone increases the expression of Beclin1, Atg7, Atg8, and LC3 proteins but decreases the expression of Bcl-2, leading to the promotion of initial autophagy by contributing to the formation of autophagosome. Furthermore, the inductive effect of autophagy by amentoflavone reduced the senescence. In addition, the levels of p53 and SIRT1 proteins were increased in the presence of amentoflavone. Therefore, these results suggest that amentoflavone increases the survival rate of cells by the induction of autophagy, which is expected as a potential candidate inhibiting various diseases related to autophagy and cell aging. 
